# Disruption of the RNA modifications that target the ribosome translation machinery in human cancer

**DOI:** 10.1186/s12943-020-01192-8

**Published:** 2020-04-02

**Authors:** Maxime Janin, Laia Coll-SanMartin, Manel Esteller

**Affiliations:** 1 Josep Carreras Leukaemia Research Institute (IJC), Badalona, Barcelona, Catalonia Spain; 2 Centro de Investigacion Biomedica en Red Cancer (CIBERONC), Madrid, Spain; 3 Physiological Sciences Department, School of Medicine and Health Sciences, University of Barcelona (UB), Barcelona, Catalonia Spain; 4 Institucio Catalana de Recerca i Estudis Avançats (ICREA), Barcelona, Catalonia Spain

**Keywords:** Ribosomal RNA, Transfer RNA, Translation, Human cancer, Epitranscriptomics

## Abstract

Genetic and epigenetic changes deregulate RNA and protein expression in cancer cells. In this regard, tumors exhibit an abnormal proteome in comparison to the corresponding normal tissues. Translation control is a crucial step in the regulation of gene expression regulation under normal and pathological conditions that ultimately determines cellular fate. In this context, evidence shows that transfer and ribosomal RNA (tRNA and rRNA) modifications affect the efficacy and fidelity of translation. The number of RNA modifications increases with the complexity of organisms, suggesting an evolutionary diversification of the possibilities for fine-tuning the functions of coding and non-coding RNAs. In this review, we focus on alterations of modifications of transfer and ribosomal RNA that affect translation in human cancer. This variation in the RNA modification status can be the result of altered modifier expression (writers, readers or erasers), but also due to components of the machineries (C/D or H/ACA boxes) or alterations of proteins involved in modifier expression. Broadening our understanding of the mechanisms by which site-specific modifications modulate ribosome activity in the context of tumorigenesis will enable us to enrich our knowledge about how ribosomes can influence cell fate and form the basis of new therapeutic opportunities.

## Introduction

Nucleotide modifications arise in most classes of RNA and more than 160 modifications are known [1]. Initial genetic studies in yeast and *E. coli* demonstrated that rRNA modifications play an important role in ribosome function [2–4], supported by further research in the area in the early 2000s [5]. Thenceforth, various ribosomal and transfer RNA modifications have been found to participate in determining cellular fate in cancer [6, 7]. For rRNA, modifications usually have an effect on early biogenesis steps, but the catalytic function of the ribosome in translation can also be subtly modulated by specific posttranscriptional modifications and selectively translate certain types of messenger RNA [8], such as the case for the rRNA methyltransferase NSUN5 [9]. Nevertheless, translational changes in the context of human cancer have been poorly studied, with most of the studies done describing how the expression of RNA modifiers could inform patient diagnosis or prognosis [10–14]. This lack of research can be explained in part by the great investment in effort and resources required to undertake a thorough study of an rRNA or tRNA modification —from cellular model construction and validation, through RNA-sequencing to visualize the transcriptome, to ribosome profiling and SILAC— in order to appreciate the differential translational efficiency of specific messengers, also known as translatomics [15]. In parallel, other methods are used to evaluate global protein synthesis, including the O-propargyl-puromycin assay and isotope pulse labeling with S35-labeled amino acid [16].

**Fig. 1:**
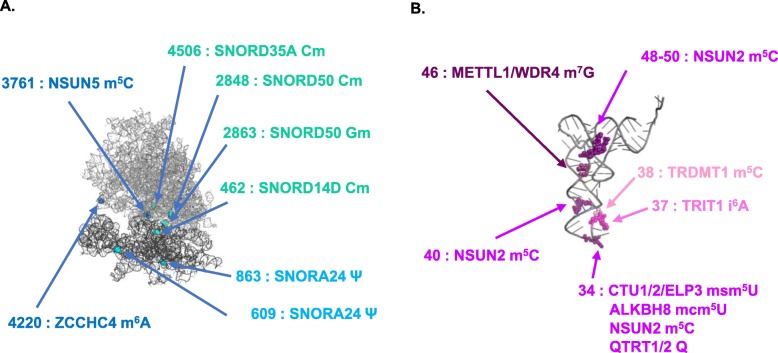
Positions of the various RNA modifications implicated in translation in human cancer in **a** the ribosome structure and **b** the transfer RNA. Structures were designed with PyMOL using 4UG0 and 5WWT identifiers from PDB

Alterations in the expression levels of few RNA modifiers of tRNA and rRNA have been linked to cancer as well as other human diseases [17–20]. However, the consequences of changes affecting the core machinery of protein translation are rarely explained. In the context of human cancer, after a brief description of the type of RNA and the machineries involved, we summarize the modifications that have been functionally characterized (Fig. 1; Tables 1 and 2). We also discuss which paths look promising in cancer therapy and provide a perspective on the possibilities for further deciphering the complex language of RNA modifications.

**Table 1:** Modifications of ribosomal RNA that affect translation in human cancer

Modification	Modifier	Cancer	References
**Nm**	Fibrillarin	Breast cancer	[21, 22]
**Nm**	SNORD50	Colon cancer	[23]
**Nm**	SNORD14D SNORD35A	Leukemia	[24]
**Ψ**	Dyskerin	Pituitary adenoma, breast cancer	[25, 26]
**Ψ**	SNORA24	Hepatocellular carcinoma	[27]
**m**^**6**^**A**	ZCCHC4	Hepatocellular carcinoma	[28]
**m**^**5**^**C**	NSUN5	Glioma	[9]

**Table 2:** Modifications of transfer RNA that affect translation in human cancer

Modification	Modifier	tRNA localization	Cancer	References
**m**^**5**^**C**	NSUN2	Cytosol & Mitochondria	Breast cancer	[29–33]
			Head and neck Squamous Carcinoma	
			Gallbladder	
**m**^**7**^**G**	METTL1 WDR4	Cytosol	Hepatocellular carcinoma	[34–36]
**mcm**^**5**^**U**	ALKBH8	Cytosol & Mitochondria	Bladder cancer	[37–39]
**mcm**^**5**^**s**^**2**^**U**	CTU1/2 ELP3	Cytosol	Melanoma	[40–42]
			Breast cancer	
**i**^**6**^**A**	TRIT1	Cytosol & Mitochondria	Lung adenocarcinoma	[43]
**Q**	QTRT1/2	Cytosol & Mitochondria	Lymphoma and leukemia	[44, 45]
			Lung cancer	
			Ovarian carcinoma	

## Ribosomal RNA

The ribosome is a complex system that translates the nucleotide code of messenger RNA into protein in cells. In humans, this complex machinery comprises four ribosomal RNAs (the 28S, 18S, 5.8S and 5S rRNAs) that are synthesized by polymerases I and III, and 80 ribosomal proteins (RPs) transcribed by polymerase II [46], all of which are organized in a large 60S subunit and a small 40S subunit. From the transcription of the ribosomal DNA to the final export into the cytoplasm, ribosome biogenesis involves more than 400 proteins and small nucleolar RNAs (snoRNAs) [47]. Ribosome heterogeneity is the result of various changes including those in the diversity of the composition of RPs and RPs mutations, abnormalities in rRNA modifications during ribosomal biogenesis, and those due to specific endogenous or exogenous factors. This heterogeneity, which also appears between cell types, has been thoroughly reviewed by Maria Barna and colleagues [48, 49]. Changes in posttranscriptional modifications of rRNAs can influence translational fidelity, leading to nonsense suppressions or amino acid misincorporations, and modifying the preferential mode of translation initiation (i.e., CAP versus internal ribosome entry site (IRES)) of key cancer genes [50, 51]. Finally, a defect in rRNA posttranscriptional modifications may cause specific clinical syndromes and may be associated with a higher incidence of cancer, which is the case for DKC1 mutation related to X-linked dyskeratosis congenita (X-DC), and for Dyskerin overexpressed in prostate cancer [52–55].

### 2′-O-methylation (Nm) machinery and implications for human cancer

The association of snoRNAs in human cancer and their potential role in cancer diagnosis and therapy has recently been reviewed [56]. The RNA component of the 2′-O-methylation machinery is a C/D box snoRNA, an evolutionarily conserved noncoding small (70–120 nucleotide-long) nucleolar RNA [57]. The assembly and composition of C/D snoRNP complexes have been extensively reviewed [5, 19, 58], and are simply summarized here. C/D box snoRNAs catalyze the methylation of ribose residues (2′-O-methylation) and can affect every type of nucleotide (A; U; C; G and pseudouridine). It contains two short conserved sequence motifs (named C and D), located near the 5′ and 3′ ends of the snoRNA, respectively, and present in duplicate (C′ and D’). It is the most abundant rRNA modification in eukaryotes, with two, 42 and 68 sites modified in the 5.8S, 18S and 28S subunits, respectively [59]. The complex involves the methyltransferase fibrillarin (FBL), an RNA-binding protein 15.5 K (NHP2L1), and a heterodimer of two closely related proteins, NOP56 and NOP58. This complex is named C/D box snoRNP (SNORD). Modification of the expression of the different factors involved can directly influence the methylation status of the corresponding ribose.

High levels of fibrillarin (FBL) are accompanied by modifications of the rRNA methylation pattern, impairment of translational fidelity, and an increase of internal ribosome entry site (IRES)-dependent translation initiation of key cancer genes [21]. Indeed, FBL is overexpressed in prostatic neoplasia [60] and breast cancer [21, 22]. It was demonstrated that FBL expression correlates with RNA polymerase I transcriptional activity and production of ribosomal RNA, and is inversely correlated with cancer-cell doubling time [61].

Chronologically, the role of 2′-O-methylation in regulating the translation of selected mRNAs was first demonstrated through the observation of associations between altered 2′-O-methylation profiles and translation vector reporter assays in breast cancer models [21, 62] and subsequently in models in which expression of FBL was knocked down [21, 22, 50, 63].

In 2009, Belin and colleagues [62] observed that breast cancer cells with increased aggressiveness displayed non-modified global translational activity but had IRES-initiated translation, notably that of p53 mRNA, which was less efficient. They also noted that control of translational fidelity was substantially reduced. This suggests that this aggressive phenotype is associated with profound alterations of ribosomal control, leading to poorer quality control of translation in cancer cells.

The expression of FBL gene is decreased by the direct binding of the tumor suppressor transcription factor p53. In human mammary epithelial cells, the diminution or suppression of p53 expression leads to modifications in the rRNA methylation pattern at the single nucleotide level, impairment of translational fidelity, and increased IRES-dependent translation of key cancer-related genes, such as IGF-1R, C-MYC, VEGF-A, and FGF1/2 [21]. As TP53 is frequently mutated in cancer, the authors showed that the level of FBL is significantly higher in mutated breast cancer than in wild type cells. They obtained the same results in a retrospective statistical analysis. Specifically, the authors showed that the rRNA methylation pattern at 18 sites distributed along the rRNAs in functionally important domains were altered in cells with p53 knockdown, increasing the rRNA methylation status due to the increased expression of FBL. Functionally, the p53-mediated increase of FBL expression leads to an increased bypass of a premature stop-codon and the misincorporation of amino acids, demonstrating the combinatory roles of p53 and FBL in translational quality control. IRES-dependent translation was stronger, whereas the CAP-dependent mechanism showed no significant differences. The complementary experiment directly modulating FBL expression confirmed that the efficacy of IRES-dependent translation initiation is driven by rRNA methylation. Clinically, the same authors have shown that FBL overexpression is associated with tumorigenesis and poor survival in patients with breast cancer and promotes cellular proliferation and resistance to doxorubicin chemotherapy of MCF7 breast cancer cells [21].

Thereafter, Su and colleagues found that FBL increased expression in association with an increasing abundance of a cluster of snoRNAs. The decrease of FBL had an anti-tumorigenic effect in vitro and in vivo, demonstrating the importance of the controlled regulation of rRNA modifications [22]. Complementary to the study of Marcel et al. [21], they revealed that FBL overexpression suppresses the p53 response to stress. As p53 mutation is relatively uncommon in breast cancer (around 20% of cases), this mechanism of FBL overexpression/p53 stress response suppression is thought to be an alternative option for p53 inactivation [22]. First, the authors showed that p53 protein stability was increased upon FBL knockdown and p53 mRNAs were enriched in polysomal fractions in these cells. They found that the level of IRES translation of p53 and other transcripts was significantly higher in FBL knockdown cells and that this mechanism is driven by the IRES trans-acting factor PTB in the context of nucleolar stress [22].

More recently, in 2017, Erales et al. characterized 2′-O-methylation in HeLa cells using a RiboMethSeq approach to quantitatively and qualitatively determine the modulation of the methylation pattern when FBL is knocked down [63]. Upon FBL knockdown, the frequency of 2′-O-methylation decreased at almost all sites, but not statistically significantly for all of them, and with differences among sites indicating site-specific regulation of 2′-O-methylation by FBL. Structurally, the affected sites were distributed throughout the ribosome structure, including functionally important regions like the A and P sites, the intersubunit bridges and the peptide exit tunnel, but in contrast, methylation sites close to the PTC and the decoding center within 18S rRNA were not affected. FBL knockdown reduced global protein synthesis, as shown by puromycylation assay and isotope pulse labeling with [^35^S]-labeled amino acids. At the mRNA-specific translational level, the authors used ribosome profiling of HeLa cells expressing an inducible FBL shRNA or control shRNA. They identified few translationally altered genes upon FBL knockdown [63]. Using a bicistronic reporter, they saw that knockdown of FBL induced a decrease in IRES-dependent translational initiation of FGF1, IGF-1R and the type II encephalomyocarditis virus (EMCV) IRES, but not of VEGFA IRES. Luciferase activity/mRNA ratios, which give an indication of the translation efficiency, showed a decrease in CAP-dependent translation, and a stronger decrease in the IRES-dependent one.

Fibrillarin affects ribosomal RNA 2′-O-methylation almost globally. Nevertheless, a few studies have identified particular snoRNA C/D boxes as site-specific writers with translational consequences in human cancer.

SNORD50 methylates rRNA at positions 28S-C2848 and 28S-G2863 in humans. Variation in SNORD50 expression has been observed in colon cancer, breast carcinoma, prostate cancer and, finally, B-cell lymphoma. Historically, the SNORD50 host gene was found by Tanaka in 2000 at a chromosome breakpoint in a human B-cell lymphoma [64]. Further studies have demonstrated an association between SNORD50 and clinical outcomes [65–67].

Regarding colon cancer, Pacilli and colleagues in 2013 studied the expression of SNORD50 in cell lines and tumors. They found that the level of SNORD50 expression was lower in tumors compared with normal counterparts, the difference being significant in a subgroup of low-stage tumors. They also found a significant association between the level of SNORD50 expression and tumor stage [23]. Notably, they showed that SNORD50 expression is inversely correlated with cellular proliferation, highlighting the fact that ribosome biogenesis requires rRNA modifications as part of the process. The overexpression of SNORD50 in HTC116 successfully increases both SNORD50 and C2848 methylation. They observed a general decrease in CAP-dependent translation and an increase in IRES-dependent translation for three IRESs: CrPV, HCV, and c-Myc, while EMCV IRES translation was not affected by changes in the methylation of this specific site [23]. Interestingly, the literature suggests that SNORD50 expression is inversely correlated with cellular proliferation and tumor progression, results that are not fully consistent with the oncogenic role fibrillarin and methylation activity play in tumors. Thus, it is possible that it is not the increase of methylation, but the aberrant 2′-o-methylation of specific positions which may be associated to different oncogenic effects.

In 2016, Zhou et al., studied an underlying mechanism of self-renewal that drives leukemogenesis. This study is an excellent example of workflow characterizing epitranscriptomic modifications in human malignancies. The authors identified a pathway that links the chimeric oncogene protein AML1-ETO to enhanced snoRNA functions via the amino-terminal enhancer of split (AES) and DDX21 interaction [24]. They showed that deletion of two snoRNAs (SNORD14D, which methylates the 18S-C462 site, and SNORD35A, which methylates the 28S-C4506 site close to the PTC) suppresses the clonogenic potential of leukemia cells in vitro and delayed leukemogenesis in vivo. Briefly, they demonstrated that AES is essential for AML1-ETO-induced self-renewal of leukemia cells in vitro and in vivo. Also in the AML1-ETO cells, snoRNAs were decreased by AES suppression. Indeed, microarray-based gene expression profiling in a comparison of fetal liver cells overexpressing AML1-ETO from mice wild-type (WT) for AES (Aes+/+) or Knockout (KO) (Aes f/f) revealed that 20 of the top 100 candidates were snoRNAs, all of which were severely reduced following AES deletion, and preferentially C/D box snoRNAs. Conversely, forced expression of AES in fetal liver cells induced snoRNA expression. At this point, they mapped the pseudouridylation to evaluate the impact of AES in rRNA modification and AES depletion weakly affected pseudouridylation in these cells. However, two 2′-O-methylation sites guided by SNORD43 and SNORD32A (methylating 18S-C1703 and 18S-G1328, respectively) showed decreased methylation. In line with a decrease in snoRNA expression, cell size was also significantly smaller in AES KO compared with WT. In AML1-ETO-induced expression cells with AES KO and a shRNA AES knockdown cell model, OP-Puro incorporation was significantly reduced, revealing a reduction in translation efficiency following AES depletion in AML1-ETO-transduced cells [24]. Depletion of NOP58 (a component of the C/D box complex) reduced C/D box snoRNA levels and rRNA methylation. Moreover, the colony formation of various leukemia cell lines was significantly reduced, suggesting a crucial role for rRNA 2′-O-methylation in clonogenicity. Knocking out six SNORDs showed that reduced levels of expression of SNORD34, SNORD35A and SNORD43 impaired clonogenic growth in the cell model, and curiously, depletion of SNORD14D reduced colony formation without affecting 18S-C762 methylation. Depletion of SNORD14D, SNORD34 (which methylates 28S-U2824), SNORD35 or SNORD43 resulted in a reduction of cell size and protein synthesis. In the context of AML1-ETO, knockout of SNORD14D or SNORD35A reduced colony formation in MV4–11 cells and delayed leukemogenesis in immunodeficient NSG mice. These results indicate that different snoRNAs have distinct roles and that some of them are important for leukemogenesis beyond AML1-ETO-induced leukemia. As expected, fibrillarin overexpression in AES-knocked-down cells increased 2′-O-methylation at various sites and promoted ribosome activity, implying that the functions of AES in AML1-ETO cells depend on both snoRNA regulation and rRNA methylation. These results are consistent with the oncogenic role fibrillarin plays in solid tumors [24].

### Pseudouridylation (Ψ) machinery and implications for human cancer

Pseudouridylation is the most common modification in RNA and the second modification found in rRNA after 2′-O-methylation. There are approximately 106 pseudouridines in rRNA clustered close to functionally important sites [59]. These modifications are driven by a snoRNA H/ACA box or by a pseudouridine synthase (PSU). PSUs recognize substrate and catalyze the isomerization reaction of uridine to pseudouridine without using any cofactors. They are classified into six families [68]. The catalytic center involves aspartate as the nucleophile for all PSUs, even in cases where the mechanism of isomerization is poorly understood [68].

RNA-dependent pseudouridylation involves an H/ACA box RNA that complexes with a set of proteins. Box H/ACA RNAs are noncoding RNAs that fold into a hairpin–hinge–hairpin–tail secondary structure. The hinge and tail regions contain evolutionarily conserved box H with the consensus sequence “ANANNA” (N for any type of nucleotide) and the trinucleotide Box “ACA,” respectively. The two hairpins contain an internal loop known as the pseudouridylation guide pocket, which has a short specific sequence complementary to the substrate RNA. The guiding pockets recognize the sites of modifications through Watson–Crick base-pairing interactions with substrate RNAs, thereby positioning the uridine to be modified at the base of the upper stem and leaving it unpaired. This brings the target site between 13 and 16 nucleotides upstream of either box H or box ACA [69, 70]. The core proteins are dyskerin (encoded by DKC1 gene), glycine–arginine-rich protein 1 (GAR1), non-histone protein 2 (NHP2), and nucleolar protein 10 (NOP10) [69, 71]. Dyskerin is the protein that exhibits the enzymatic activity within this complex, which is known as H/ACA snoRNP. This enzyme has two distinct functions: the pseudouridylation necessary for rRNA processing, and the stabilization of the telomerase RNA component that is essential for telomerase activity. Similar to fibrillarin for C/D box RNPs, DKC1 or dyskerin regulation affects its rRNA epitranscriptomic activity. In 1998, Heiss et al. identified the mutation responsible for X-linked recessive dyskeratosis congenita in a gene named XAP101 [52]. This disease is characterized by bone marrow failure, skin abnormalities, and an increased susceptibility to develop cancer [72]. Pseudouridylation is thought to play a role in translation, modulating the interactions between tRNA, rRNA and mRNA. The influence of modified nucleosides on the local structure of the antisense loop is essential for the proper binding of tRNA to the ribosome [73]. Maintenance of the proper conformation of the three anticodon residues helps foster correct codon–anticodon interactions. This may increase translational accuracy by reducing the rate of peptide bond formation, thereby allowing more time to reject incorrect codon–anticodon pairs [73].

In 2006, Montanaro and colleagues showed that, in human breast cancer, low levels of expression of dyskerin, as determined by immunohistochemistry and RT-PCR, were associated with a better outcome [74]. The same year, Yoon et al. were the first to report the influence of dyskerin expression on translation, although this time it occurred in X-linked dyskeratosis congenita (X-DC) [75]. Using a knocked-in DKC1 point-mutation mouse model, they saw that reduced rRNA pseudouridylation did not affect total protein synthesis. They then analyzed the translationally active ribosome-associated mRNAs from steady state and activated primary splenic lymphocytes, one of the hematopoietic lineages affected in X-DC. They identified three out of 1500 mRNAs whose expression was specifically decreased in polysome-associated fractions for DKC1^m^ lymphocytes: the p27 tumor suppressor, the anti-apoptotic proteins XIAP (X-linked Inhibitor of Apoptosis Protein), both of which harbor IRES elements, and Bcl-xL showed a significant decrease in their association with polysomes in DKC1^m^ cells compared with wild-type cells [75]. The decrease was observed only at the protein level, where a specific translation in DKC1 cells was revealed. Yoon et al. confirmed IRES-dependent translation of p27 and XIAP and also established that BCL-XL contains a previously unreported IRES element. In 2010, the same group, headed by Davide Ruggero, showed that DKC1 is mutated in pituitary adenoma and that this genotype causes a defect in pseudouridylation activity and reduced expression of p27 [25]. They observed that this translation was reduced in the pituitary of DKC1^m^ mice. DKC1^m^/p27^+^/^−^ mice developed a similar pituitary malignancy to that of p27^−^/^−^ mice. Finally, they found a DKC1 mutation in patients with pituitary adenoma that drastically reduced DKC1 expression and pseudouridylation, but also brought about a reduction in p27 expression. Therefore, DKC1 is a tumor suppressor that controls translation by its direct role in rRNA modifications [25]. In parallel, they also established that p53 IRES-dependent translation is impaired in DCK1^m^ cells during oncogene-induced senescence in X-DC [76]. Normally, during this senescence transition, IRES translation is facilitated, promoting the translation of p53. This mechanism in X-DC is close to that controlling fibrillarin overexpression, which inhibits p53 expression [21]. In 2013, Rocchi et al. found that angiogenesis in human breast epithelial cells was promoted by enhanced VEGF-IRES-mediated translation associated to the lack of dyskerin [26].

A H/ACA box RNP has been identified as playing a role in tumorigenesis by regulating translation. A recent study by McMahon and colleagues has shown the regulation of SNORA24 in human hepatocellular carcinoma and its collaboration with RAS mutation to promote cancer [27]. SNORA24 drives two pseudouridylations in the 18S rRNA at positions 609 and 863. Ribosomes lacking the corresponding modifications exhibited perturbations in the aminoacyl-transfer RNA (aa-tRNA) selection and altered pre-translocation ribosome complex dynamics [27].

Further research is needed to fully understand the interplay between genetic alterations, regulation of epitranscriptomics and translation in human tumorigenesis.

### Other modifications of ribosomal RNA affecting translation in human cancer

Only two recent studies have identified other types of rRNA modification impairment that influence translation in human cancer.

The first, by Ma and colleagues in 2018, identified the N^6^-methyladenosine ZCCHC4 as being responsible for modifying the rRNA at position 4220, and this methyltransferase is overexpressed in human hepatocellular carcinoma [28]. This position is not in a functionally important region but, as seen before, changes in translation are not linked to changes of modifications in these regions. Using a luciferase reporter and then l-homopropargylglycine-derived metabolic labeling of newly synthesized proteins, the authors showed that ZCCHC4 KO in hepG2 cells displayed a lower level of global translation than in WT. After a sucrose density gradient and polysome-associated mRNA sequencing, they suggested that loss of m^6^A methylation should result in a reduction of the tight control of translation in mRNA populations, and might increase the translation of a subset of mRNAs. Finally, gene ontology indicated that genes affected by ZCCHC4 are involved in membrane protein targeting, the mRNA catabolic process, ER localization, and translation initiation. We recently studied the role of NSUN5, an m^5^C methyltransferase, in human gliomagenesis [9]. NSUN5 methylates the rRNA at position 28S-C3761 (old nomenclature 3782) and influences global translation, since, under conditions of stress, reduced OP-puro and H3-leucine incorporations were observed in cell lines depleted in NSUN5 [9]. This condition also leads to a specific translation in response to stress, as determined for some proteins regulated only at the translational level. We chose to validate NQO1 as a relevant candidate for therapeutic targeting [9].

Other rRNA modifiers have been linked to human cancer, but the underlying mechanisms driving translational change require further investigation. NAT10, for example, is a N-acetyltransferase involved in histone and microtubule modification, pre-rRNA processing, but also in the acetylation of rRNA at positions C1337 and C1842, and the acetylation of leucine/serine tRNA [77, 78]. NAT10 has been found upregulated in hepatocellular carcinoma (HCC) [79], ovarian cancer [80] and AML [13], and correlates with poor survival of patients. This upregulation also promotes metastasis of HCC cells [81, 82]. Also, sub-cellular redistribution of NAT10 in colorectal cancer is able to promote cell motility through modifying cytoskeleton dynamics [83]. Another modifier, NOP2 (also named NSUN1 or p120), a probable rRNA methyltransferase for position C4447 of the human 28S RNA [84], has been described as overexpressed in lung adenocarcinoma [14, 85]. In HCC, Wang and colleagues have described that LncRNA-hPVT1 stabilizes NOP2, thereby increasing cell cycle rate, and promoting both cell proliferation and the acquisition of stem cell-like properties [86]. The cytosine methylated by NOP2 is part of the A-loop of the peptidyl transferase center. This is a critical position within an area responsible for the initial interaction with the incoming peptidyl-tRNAs, as well as the presentation of tRNAs in the correct conformation for the optimal peptidyl transfer reaction [84]. This mechanism could have major impact on protein synthesis in cancer cells with NOP2 expression changes. Overall, the role of rRNA modifications in the tumorigenic mechanisms driven by these two modifiers will need further investigation.

In summary, few studies have clearly identified changes in translation in human cancer, but there are enough of them to reveal the importance of this regulation in human tumorigenesis (Fig. 1a; Table 1).

## Transfer RNA

Transfer RNAs are small RNAs that carry amino acids to ribosomes that translate an mRNA. This allows amino acid incorporation into the synthesized peptide according to the corresponding codon. In humans, tRNAs can carry up to 21 different amino acids. tRNAs present five functional arms or loops. The aminoacylation arm is where the aminoacyl synthase incorporates the corresponding amino acid, whereas the anticodon loop is the region containing the three nucleosides that pair with the mRNA codon. There are also the D-loop, so called because of the dihydrouridines it contains, the T-loop for the conserved TψC sequence, and the variable arm, whose length differs in each tRNA [87].

tRNAs are synthesized by RNA polymerase III and all pre-tRNAs undergo 5′ and 3′ end processing, and addition of CCA to the processed 3′ end. Some tRNAs also require intron cleavage and subsequent ligation. tRNA biogenesis occurs in the nucleus and mitochondria, depending where they are codified. Those that are synthesized in the nucleus are exported to the cytoplasm as a final step [88, 89]. Aminoacylation is the covalent attachment of amino acids to their corresponding tRNA, the reaction being catalyzed by aminoacyl-tRNA synthetases. There is at least one enzyme for each amino acid, and they can function in the cytoplasm, the mitochondria, or in both locations [90]. Throughout this process, tRNA can undergo nucleoside modifications, some of which are introduced into all tRNAs, whereas others are tRNA-specific. Modifications can also vary between cytoplasmic and mitochondrial tRNA.

RNA polymerase III, aminoacyl-tRNA synthetases and other proteins involved in tRNA maturation steps are known to be altered in a wide range of tumors. This can be explained by the need for tRNA availability and translation efficiency to supply the higher level of protein synthesis that occurs in cancer cells [91, 92].

During different steps of their maturation, modifications are anchored to tRNA, allowing the correct folding of their secondary (cloverleaf) and tertiary (L-shaped) structures through correct base-pairing, codon recognition and binding. These ensure the fidelity of translation, structural stability and integrity [29, 37, 93]. Disturbances in the regulation of these steps can create opportunities for cancer to arise.

### Transfer RNA modifications in human cancer

#### 5-methylcytidine (m^5^C)

5-methylcytidine is incorporated at different positions of tRNA by NSUN2, NSUN3, NSUN6, and TRDMT1.

NSUN2 and TRDMT1 are the best functionally characterized epitranscriptomic enzymes. TRDMT1 methylation prevents tRNA cleavage [94, 95]. tRNA fragment accumulation and loss of cytosine-5 methylation in tRNAs was associated to decreased ribosome density in mRNAs and increased ribosome density in 5′ UTRs [96]. In mice, methylation also confers stability to their tRNA substrates as loss of 5-methylcytidine causes tRNA degradation, and loss of Trdmt1 and Nsun2 leads to a global decrease of protein synthesis [29]. Upregulation of NSUN2 expression was identified in one-third of primary breast tumors and breast cancer cell lines. Also, high levels of NSUN2 expression were detected in head and neck carcinoma. In these studies, this aberrant expression significantly increases mortality [30–32]. Moreover, the oncogene RLP6 has been found to partially regulate NSUN2 translation and so tumorigenesis function in human gallbladder carcinoma [33].

#### m^7^G

METTL1 and WDR4 are both necessary for the introduction of the 7-methylguanosine at position 46 [34]. In 2018, Lin et al. have reported that METTL1 knockdown reduces the m^7^G fraction, which causes tRNA destabilization and ribosome pausing at m^7^G tRNA-dependent codons that leads to a decrease in translation [35]. Overexpression of METTL1 in hepatocellular carcinoma has been shown to promote cell proliferation and migration, and to lead to poor prognosis in HCC [36].

#### mcm^5^U

5-methoxycarbonylmethyluridine (mcm^5^U) is a common modification in the wobble position. ALKBH8, with TRM112 as a partner, catalyzes the methylation of 5-carboxymethyluridine (cm^5^U) to mcm^5^U [37, 38]. tRNA^Sec^ is one of the targets of ALKBH8, this tRNA transfers the amino acid selenocysteine during selenoprotein translation. A large number of selenoproteins are involved in the detoxification of ROS. It has been shown that ALKBH8 deficiency causes a decrease in selenocysteine protein translation and consequently ROS stress that triggers oxidative DNA damage [38, 97]. It has been reported both in vitro and in vivo that ALKBH8 promotes the expansion, survival and invasion of bladder cancer [39, 98]. Cancer cells use AKBH8 expression as an antioxidant system against the ROS stress generated during tumorigenic transformation [97].

Msm^5^U is necessary for the formation of some derivatives, such as 5-methoxycarbonylmethyl-2-thiouridine (mcm^5^s^2^U). CTU1, CTU2 and ELP3 are the thiolation effectors with URM1 as the sulfur donor [37, 40, 99]. The mcm^5^s^2^U complex is heavily implicated in cancer. On one hand, its enzymes levels have been detected at higher levels in human tumor biopsies and in cultured melanoma cells with BRAF^V600E^ mutation. An in silico analysis revealed that this kind of melanoma also has high levels of hypoxia-induced factor 1α (HIF1α), which is known for its involvement in tumorigenesis progression. The mRNA sequence of HIF1α is rich in U34 codons that require tRNA modification for correct translation. Thus, U34-related enzyme overexpression triggers increased HIF1α translation and thereby cancer transformation [41]. Similarly, U34-related enzymes were found to be overexpressed in breast cancer patients. The IRES trans-acting factor (ITAF) protein DEK has U34 codon-dependent translation regulation. DEK promotes the translation through the IRES sequence of LEF1, whose expression is associated with a higher risk of metastases [42].

### Isopentenyladenosine (*i*^*6*^*A*)

TRIT1 introduces a dimethylallyl pyrophosphate at position 37, generating an isopentenylated adenosine in both cytoplasmic and mitochondrial tRNA [100–102]. In particular, it has been related to selenoprotein expression since tRNA^Sec^ is one of its targets [103]. Expression of some selenoproteins are influenced by TRIT1 depletion [43]. Spinola et al. observed downregulation of TRIT1 expression in lung adenocarcinoma patients and noted that expression of TRIT1 in a lung adenocarcinoma cell line diminishes tumorigenesis. Its tumor suppressor effect is indicated by the role of some selenoproteins in diminishing oxidative stress [104]. However, as demonstrated in the case of ALKBH8, which modifies mcm^5^U34, cancer can also use selenoproteins to maintain oxidative stress at sublethal levels. Similarly, i^6^A is needed for ALKBH8 modification to take place [43].

### Queuosine (*Q*)

Queuosine (7-(((4,5-cis-dihydroxy-2-cyclopenten-1-yl)amino)methyl)-7-deazaguanosine) is a derivative of the 7-deazagunine located in the wobble position. This modification is introduced by the tRNA-guanine transglycosylase (TGT) heterodimer formed by QTRT1 (catalytic subunit) and QTRT2 [44]. Eukaryotes cannot synthesize queuine but they obtain it from gut microbes or food. Tuorto et al. suggested that G34-containing tRNAs cause a translational delay that induces an accumulation of misfolded proteins and hence, endoplasmic reticulum stress and an unfolded protein response. In order to avoid this situation, Q modification prevents the elongation from slowing down [105]. Reduced Q content has been detected in a wide range of cancers [45].

This overview of the modifications affecting transfer RNAs (Fig. 1b; Table 2) summarizes the characteristics that influence cellular fate and that can drive tumorigenic processes.

## Conclusions

Regulation of translation is crucial for cell proliferation and cell fate in human malignancies. There is increasing evidence that epitranscriptional modifications affect translation by modifying the affinity or the stability of mRNAs, tRNAs, or the interactions between them inside the ribosome.

**Fig. 2:**
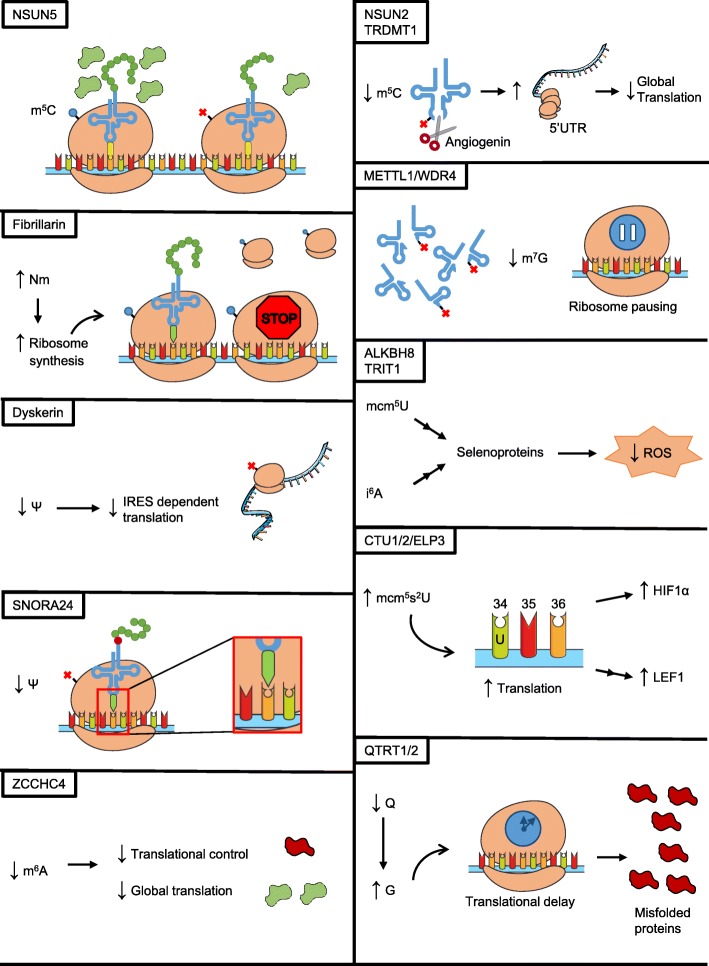
Schematic representation of translational effects triggered by RNA modification defects at rRNA and tRNA in the context of cancer cells

Here, we have reviewed the various modifications of transfer and ribosomal RNAs that are known to have consequences for translation in human cancer (Fig. 2). We chose to exclude modifications affecting other types of RNAs and alterations in the expression and activity of specific translation factors, not only because these have already been extensively reviewed [106, 107], but also to present a consistent overview of the role of RNA modifications in the core functions of translation.

The role of epigenetics in cancer is now very firmly established, to the extent that corresponding therapies exist. However, the recent expansion of our knowledge about epitranscriptomics has not yet resulted in any clinical applications, although there is evidence of prognostic markers and possible treatments. In this sense, the exhaustive characterization and classification of RNA modifiers in each relevant cell type as tumor suppressor or oncogene will provide us with tools to identify new targets and design specific treatments that disrupt the carcinogenic pathways [108, 109]. Certainly, it is very important to identify the cellular background, as for a specific cancer type, different environments can be beneficial or lead to apoptosis [110].

Studies highlight that fibrillarin is frequently overexpressed in human cancer, and its expression controls the fine-tuning of ribose 2′-O-methylation of the rRNA in human cancer. FBL depletion leads to the regulation of IRES translation globally, but some specific mRNAs are altered in a different way, such as p53, whose expression increases when FBL is knocked down, and also revealed by ribosome profiling [21]. Further studies would certainly confirm the qualitative and quantitative role of FBL in human cancer translation and would produce substantial information about identifying the downstream molecular and cellular functions affected by this mechanism. Cellular context is a critical factor as a stress influence on the translational response to FBL modulation [21]. Clinically, fibrillarin has not yet been exploited as an oncological target, but a recent review emphasizes its potential as a therapeutic target that could lower the genotoxic effects of anti-cancer treatment. This could be achieved by exploiting the association between low levels of FBL expression and better breast cancer survival, and its functions in ribosome biogenesis and p53 regulation [111]. Other 2′-O-methylation modifiers have shown therapeutic potential, such as SNORD44 in colorectal cancer and SNORD47 in glioblastoma [112, 113].

Few studies have highlighted the relevance of dyskerin in driving cancer development. Their findings can explain in part the increased risk of cancer observed in X-DC. In some other studies, the translational impact of pseudouridylation defects is not clear. This is the case for SNORA42 in lung cancer [10], in which SNORA42 is overexpressed in non-small cell lung cancer and its suppression inhibits cell growth, proliferation and tumorigenicity of cancer cells by inducing p53-dependent apoptosis. These results were partially confirmed 2 years later [114], but a translational shift driven by SNORA42 remains to be conclusively demonstrated. Strikingly, Bellodi et al., found that SNORA42 expression is increased in *DKC1*(ΔL37)-mutated lymphocytes, which raises the possibility that certain snoRNAs may be selectively increased as a compensatory mechanism for perturbations in subsets of H/ACA snoRNAs [115]. They also observed an unexpected heterogeneity of expression of the H/ACA snoRNAs in cells of patients with X-DC harboring distinct DKC1 mutations. This is in line with the unexpected 2′-O-methylation alteration observed in X-DC. Very recently, a study linked the rRNA 2′-O-methylation alteration caused by NPM1 mutations to the development of congenital dyskeratosis [116]. These results demonstrate the complex etiology of such diseases and how a common phenotype can arise as the result of different alterations.

Modifications can directly influence the level of tRNAs by modulating their stability. The by-products formed by angiogenin cleavage regulate translation in human cancer. There are many examples in the literature of efforts that have been made to improve our understanding of the subtle influence of proteomic modifications and the consequent tumorigenesis. Another aspect emerging from these studies is the difficulty of appreciating the enzyme-site-specific role. Indeed, this is the case for ribosomal RNA modifiers also, lots of enzymes have a panel of activities, and the modulation of its expression can have side effects that also play a role in translational changes. For example, TRIT1 and ALKBH8 functions are deeply related as mcm^5^U have been described as i^6^A dependent, although they are described as a tumor suppressor and oncogene respectively, some hypotheses exist as other functions of the genes but more research are needed to clarify it. This is also the case for NSUN5, we related the translational changes to the modification on the rRNA as it logically seems to have the most direct role on ribosome function. Nevertheless, considering the clinical implications of these modifiers, it is certain that the global phenotype takes precedence over the single modification. A nuance is the relevance of a cancer-specific translation in drug development and therapeutic opportunities [117]. For example, in 1990, the discovery that eIF4E overexpression could drive tumor initiation has led to the development of strategies to decrease its expression. To go further, ribosomal RNA modifications can lead to antibiotic resistance in yeast, so antibiotic-like drugs could specifically target the altered RNAs in cancer. In this regard, in 2013 Begley and colleagues found that tRNA methyltransferase 9-like protein (hTRM9L) was downregulated in breast, bladder, colorectal, cervix and testicular carcinomas and this downregulation was associated with altered tRNA modification levels [118]. Importantly, hTRM9L-deficient tumors were highly sensitive to aminoglycoside antibiotics [118]. Regarding tRNAs, NSUN2 overexpression indicates a shorter overall survival in head and neck carcinomas [32]. Another example is ALKBH8, which is crucial in bladder cancer transformation. It has been proposed that an intravesical injection of ALKBH8 siRNA may reverse the invasive character of the neoplasm in patients [38, 98]. It has also been described that a deficit of ALKBH8 increases the sensitivity to DNA-damaging agents and could complement the previous strategy [38]. Moreover, Rapino et al. proposed that targeting wobble uridine 34 (U34) tRNA (U34 enzymes) can avoid the resistance to Vemurafenib in BRAFV^600E^ melanoma [41].

RNA modifications have a promising future. The relatively small number of studies, whether considering tRNA or rRNA, that completely describe the translational role of a specific modification in this context highlights the significant gap that remains to be filled if we are to better understand the complex mechanism of tumorigenesis (Fig. 1; Tables 1 and 2). In this puzzle, the addition or removal of a piece (RNA modifications) can effectively change the final picture.

## Data Availability

Not applicable

## Abbreviations

FBL: Fibrillarin
IRES: Internal ribosome entry site
i^6^A: Isopentenyladenosine
ITAF: IRES trans-acting factor
m^5^C: 5-methylcytidine
mcm^5^U: 5-methoxycarbonylmethyluridine
m^7^G: 7-methylguanosine
PSU: Pseudouridine synthase
Q: Queuosine
RPs: Ribosomal proteins
rRNA: Ribosomal RNA
SILAC: Stable Isotope Labeling by/with Amino acids in Cell culture
SNORD: C/D box snoRNP
snoRNAs: Small nucleolar RNAs
TGT: tRNA-guanine transglycosylase
tRNA: Transfer RNA
X-DC: X-linked dyskeratosis congenita
